# Family planning knowledge, attitude and practice among Rohingya women living in refugee camps in Bangladesh: a cross-sectional study

**DOI:** 10.1186/s12978-022-01410-0

**Published:** 2022-05-02

**Authors:** Md. Abul Kalam Azad, Muhammad Zakaria, Tania Nachrin, Madhab Chandra Das, Feng Cheng, Junfang Xu

**Affiliations:** 1 Department of Communication and Journalism, University of Chittagong, Chattogram, 4331 Bangladesh; 2 Department of Communication, University of Louisiana, Lafayette, 70504 USA; 3 Vanke School of Public Health, Tsinghua University, Beijing, 100084 China; 4 Institute for Healthy China, Tsinghua University, Beijing, 100084 China; 5 Center for Health Policy Studies, School of Public Health, Zhejiang University School of Medicine, Hangzhou, 310058 China

**Keywords:** Family planning knowledge, Family planning attitude, Family planning practice, Contraceptive use, Rohingya displaced women, Refugee camps

## Abstract

**Background:**

Considering the high risk of maternal morbidity and mortality, increased risks of unintended pregnancy, and the unmet need for contraceptives prevalent among the Rohingya refugees, this study aims to explore the knowledge, attitude, and practice (KAP) of family planning (FP) and associated factors among Rohingya women living in refugee camps in Bangladesh.

**Methods:**

Four hundred Rohingya women were interviewed. Data were collected using a structured and pretested questionnaire, which included study participants’ socio-demographic characteristics, access to FP services, knowledge, attitude, and practice of FP. Linear regression analysis was performed to identify the influencing factors of FP-KAP.

**Results:**

Of the 400 Rohingya refugee women, 60% were unaware that there was no physical harm brought by using a permanent method of birth control. Half of the women lack proper knowledge regarding whether a girl was eligible for marriage before the age of 18. More than two-thirds of the women thought family planning methods should not be used without the husband’s permission. Moreover, 40% were ashamed and afraid to discuss family planning matters with their husbands. Of the study participants, 58% had the opinion that a couple should continue bearing children until a son is born. Linear regression analyses found that study participants’ who have a profession, have less children, whose primary source of FP knowledge was through a physician/nurse, have had FP interventions in the camp, and talk with a health care provider on FP were found to have better FP-KAP.

**Conclusion:**

The study showed that Rohingya refugee women are a marginalized population in terms of family planning and their comprehensive FP-KAP capability was low. Contraceptives among the Rohingyas were unpopular, mainly due to a lack of educational qualifications and family planning awareness. In addition, family planning initiatives among Rohingya refugees were limited by a conservative culture and religious beliefs. Therefore, strengthening FP interventions and increasing the accessibility to essential health services and education are indispensable to improving improve maternal health among Rohingya refugees.

## Plain Language Summary

Considering the high risk of maternal morbidity and mortality, increased risks of unintended pregnancy, and the unmet need for contraceptives prevalent among the Rohingya refugees, this study aims to explore the knowledge, attitude and practice (KAP) of family planning (FP) and associated factors among Rohingya women living in the refugee camps in Cox’s Bazar, Bangladesh. Four hundred Rohingya women participated in the study. We found that Rohingya refugee women were a marginalized population in family planning and their comprehensive FP-KAP status was low. Contraceptive uptake among the Rohingya women was low due to a lack of education and family planning awareness. In addition, family planning initiatives among Rohingya refugees were limited by various traditional cultural and religious beliefs. Therefore, strengthening FP interventions and increasing accessibility to essential health services and education are indispensable to improving maternal health among refugees.

## Background

Over the last few decades, the number of stateless people who are usually identified as refugees has grown exponentially around the world. The most recent focus is Myanmar’s Rohingya diaspora, who have left their homes since 25 August, 2017 [1–3]. This influx of more than 700,000 Rohingya into Bangladesh has produced the fastest-growing refugee crisis in the world [1, 2, 4–6]. Bangladesh’s total number of unregistered refugees was about 220,000 before the recent influx [2, 7]. However, as of 31 March 2021, approximately 884,000 Rohingya refugees who are Forcibly Displaced Myanmar Nationals (FDMN) resided in 34 camps in Ukhiya and Teknaf Upazilas (sub-districts) of Cox’s Bazar District of Bangladesh [8], which have grown to become the largest and most densely populated camps in the world [9]. Among the refugees, women and children make up the majority [6, 10–12], which accounts for more than 50% [1, 13–15].

The Government of Bangladesh and development partners, including the United Nations High Commissioner for Refugees (UNHCR), United Nations Children's Fund (UNICEF), United Nations Population Fund (UNFPA) and the World Health Organization (WHO) are working together to provide humanitarian relief to the Rohingya people [4]. The Rohingya, while living in Myanmar, were deprived of nationality and fundamental rights to education and health care. These restrictions have substantially affected their knowledge of contraception and family planning [16, 17], indicating that adverse health outcomes related to maternal health may be extremely high [18]. Evidence also suggests that worldwide, forcibly displaced women and adolescent girls are experiencing intensified sexual and reproductive health (SRH) concerns, including a high risk of maternal morbidity and mortality, increased risks of unintended pregnancy, and an unmet need for contraceptives [1, 14, 19]. For example, 179 mothers die from preventable causes related to pregnancy and childbirth for every 100,000 live births in the camps [20]—nearly two-and-a-half times the global maternal mortality goal [21]. Save the Children estimated that 76,000 babies were born in the Rohingya camps in Bangladesh over the past 3 years [22]. Correspondingly, more than 60 babies were born every day in the refugee camps of Bangladesh [23].

In order to address reproductive and maternal health issues of Rohingya women and adolescent girls, humanitarian actors collaborated with the Ministry of Health and Family Welfare (MOHFW), providing basic health services including family planning (FP) programs, intrauterine devices (IUD) and implants, as well as other short-acting modern methods of FP (condoms, oral contraceptive pills, injectables) [11, 14–16, 24, 25] to increase community awareness [1, 14]. Moreover, at the community level, health workers are implementing different interventions including FP counseling sessions and community meetings with the intended population at reproductive age [1, 4, 16].

According to estimations from recent studies, the Rohingya women’s contraceptive prevalence rate (CPR) was higher than reported in 2018. A survey conducted by the International Center for Diarrhoeal Disease Research, Bangladesh (ICDDR,B) [3] exposed that contraceptive use amongst Rohingya refugees rose by 2.1 percentage points from 33.7% in 2018 to 35.8% in 2019. Increasing awareness of modern contraceptive methods among Rohingya refugees may contribute to these positive outcomes. However, challenges also exist due to cultural values, traditional misconceptions, and dogmatic beliefs towards contraceptive use among the majority of the Rohingya people [1, 4, 6, 14, 16, 17, 26–29]. The hindrances may include: the religion of Islam not permitting use of contraceptives [1, 4, 16], husbands’ disapproval of contraceptive use [1, 14, 16, 29], actively trying to fall pregnant [28, 29], the belief that lessening the number of children is a sin [1, 4, 29], the belief that a child is a gift of Allah (God) [1, 4, 16, 29], considering children as economic assets [4], considering that a large family would enable better chances of survival in refugee camps [4, 30], the belief that use of contraceptives can lead to adverse health outcomes including infertility [16], and the negative role of husbands and mothers-in-law as the two most influential decision-makers regarding contraceptive use [4]. In addition, despite various organizations’ providing FP services, Rohingya women and girls do not have adequate and equal access to these services [16]. This highlights the significance of increasing contraceptive use among Rohingya women and improving their maternal health. Under this background, we aim to analyze FP in terms of knowledge, attitude and practice and other associated factors among the Rohingya women living in Cox’s Bazar refugee camps, Bangladesh. It is hoped that this study could provide evidence for developing interventions in a coordinated and effective manner to improve maternal and child health for Rohingya women in refugee camps.

## Methods

### Study design and setting

This study used a quantitative research approach designed with a camp-based cross-sectional survey. It was conducted at Rohingya refugee Camp-4 (located at Lombashiya, Modhurchora in Kutupalong Mega area) in Cox’s Bazar, a district under the Chittagong Division, geographically the largest of the eight administrative divisions of Bangladesh. This camp was selected as the study area as it is one of the largest camps in Bangladesh.

### Participants

The population of the study consists of married Rohingya refugee women of reproductive age (18–49 years old) who had been living with their husbands at the camp and had delivered at least one child at least 1 year before the survey was conducted. A total of 32,389 Rohingya people were living in Camp-4 during the study period while the number of women was 16,968, and 7683 of them were women of reproductive age [31]. The sample size was determined using the single population proportion formula considering the following assumption: p = 50% (it was hypothesized that the percentage frequency of having better FP-KAP in the population was 50% for the estimated proportion of Rohingya women), significance level 5% (α = 0.05), Z $\frac{\mathrm{\alpha }}{2}$ = 1.96, margin of error 5% (d = 0.05) and assuming 10% non-response rate. The required sample size was 422, which is the number of individuals the research team invited to participate in the survey. Finally, a total of 400 refugee women (94.79% response rate) participated in the study. Study participants were selected following convenient sampling, due to the humanitarian context and inadequate funds. A previous study [32] also faced this methodological challenge due to the structure of the camps. In the camp, the houses were built sporadically on hills with no identification numbers. Furthermore, there was no complete list of Rohingya persons living in a particular block or camp. Registered Rohingya people were also hesitant to provide their registration numbers, making it difficult to establish a sampling frame. Due to time constraints, we were unable to compile a list of households to construct a sampling frame for simple random sampling. As a result, the study team chose convenience sampling.

### Reliability and validity of the instrument

In order to ensure the relevance of the questionnaire items with the study aims, the content validity of the questionnaire was reviewed by three experts working in the same field. Each expert reviewed the questionnaire separately and various changes were made to the questionnaire based on their recommendations. The internal consistency was also measured to check the reliability. Cronbach’s Alpha (α) values of the scale of FP knowledge, attitude, and practice suggested very good internal consistency reliability for the scales of this study. The alpha (α) value was good among knowledge-related 10 items (α = 0.84) and attitude-related 10 items (α = 0.89) and strong among practice-related 10 items (α = 0.95).

### Study variables

There were three dependent (outcome) variables in our study which included knowledge of FP, attitude towards FP, and practice regarding FP. The independent (potential predictor) variables included the respondents’ region of residence in Myanmar, age, educational status, occupation, amount of land owned in Myanmar, and number of children. We included the respondents’ educational and residential status and amount of land owned in Myanmar under the socio-demographic variables as we hypothesized that these past statuses might be an important indicator of health behavior in the current settings. Media use–related variables were respondents’ listening to radio and internet use, while access to NGO programs and health facilities included respondents’ prime source of FP knowledge, person(s) who make respondents’ SRH-related decisions, availability of NGO FP activities in camp, respondents’ participation in FP programs, visiting of clinic/health facilities, talking with a health care provider. To gain further context, we also collected information on different FP methods heard and used by the Rohingya women and the main reasons for not using a contraceptive.

### Data collection

Data collection began on October 14 and was completed on December 26, 2019. Data were collected using a pretested, structured, and facilitator-administered questionnaire. The questions used in the questionnaire were prepared based on a review of related literature. The questionnaire was developed in Bengali (Bangla) language applicable to the context in Bangladesh. It was not translated into the Rakhine/Arakanese language of the Rohingya people since it lacks an appropriate written form the majority of the Rohingya people in the camp are illiterate. The survey was guided and conducted by ten female data collectors who had graduate degrees and work experience in the Rohingya camp and were quite familiar with the study setting. The data collectors were fluent in the Rakhine/Arakanese language, which helped them explain the questions to the interviewees and understand the responses. Ten Rohingya women, who were known as the community leaders in the survey area, assisted with the data collection process in the camp. They helped build rapport and gain the trust of the participants. Thus, it is believed that the participants felt comfortable speaking openly and sharing issues in their personal life. The Rohingya women trust these community leaders, so therefore they agreed to cooperate and participate in the study [32]. All the recruited Rohingya women had experience working with their community. The interviews took place at different blocks of the camp. Before the survey, a pilot study was conducted among 40 Rohingya women to test the understandability of the survey and to ensure its comprehensiveness and consistency in providing the information needed for the study.

Despite the limitations encountered due to camp’s layout, privacy and confidentiality were ensured during the data collection process. The community leaders obtained permission from the respondents beforehand to ensure that the respondent would be free and comfortable to talk. Before starting the questionnaire interview, the purpose and confidentiality of the study were clearly explained to the respondents. Interviews were conducted in a quiet room of the house of the respective refugee women situated in a particular block of the camp where only the data collector and a community member were present. An appropriate level of privacy was able to be met whilst collecting the data as the male members of families generally undertake daily work during the day and children go to the learning centers, child-friendly spaces or madrasas (religious center to study Quran). Moreover, prior to the interview the respondents asked the other family members to go to another room so that they could talk with data collectors comfortably.

### Measurements

KAP items having 10 items for each section were designed with a five-point Likert scale. For the FP knowledge section, the score of each positive statement ranged from 1 to 5 for ‘definitely false’, ‘probably false’, ‘do not know’, ‘probably true’ and ‘definitely true’. For the FP attitude section, the score of each positive statement ranged from 1 to 5 for ‘strongly disagree’, ‘disagree’, ‘neutral’, ‘agree’, and ‘strongly agree’. For the FP practice section, the score of each positive statement ranged from 1 to 5 for ‘never’, ‘rarely’, ‘sometimes’, ‘often’, and ‘always’. The score was reversed for negative statements. The total score of FP knowledge, attitude, and practice was the sum of the score for questions under each section respectively. In order to understand the level of FP knowledge and attitude, we categorized both variables’ scores into two levels using the mean as the cut-off value. However, five scales of each section were recoded into three categories because of the low frequency at the endpoint of the scale for the percentage distribution of respondents’ responses regarding KAP.

### Statistical analysis

Descriptive statistics were used to analyze the respondents’ FP-related KAP. A linear regression analysis was performed to estimate the proportion of variance in FP knowledge, attitude, and practice based on socio-demographic, NGO, and health facility-related factors. The linear regression models included the variables with p < 0.05 in bivariate analyses (independent-samples t-test and Pearson correlations). Multicollinearity was also checked. The ANOVA values for overall FP knowledge (*F* = 64.84, *p* < 0.001), attitude (*F* = 59.56, *p* < 0.001), and practice (*F* = 170.36, *p* < 0.001) report that the regression model was a good predictor of the main outcome variables. *R*^2^ of each step was changed considerably, and *F* changes were also statistically significant (*p* < 0.001). These analyses were performed with a 95% confidence interval using SPSS 24.0. Variables with *p* < 0.05 were considered statistically significant.

## Results

### Socio-demographic characteristics of Rohingya women

Table 1 showed that of the 400 respondents, 210 (52.4%) were residents of Buthidong sub-district of Myanmar before taking shelter in Bangladesh. The mean age was 25.53 (± 6.34) years. More than half (51.8%) of them had no formal education and more than three-quarters (78%) were housewives. On average, the study participants had 4 (3.98 ± 2.60) children. Regarding media use, 233 (58.2%) listened to the radio and 103 (25.8%) used the internet. In addition, 181 (45.3%) women reported that NGO workers and health workers were their primary sources of FP-related information.

**Table 1:** Socio-demographics and other background characteristics of the Rohingya women

Variable	Number	Percentage (%)
Region of residence in Myanmar		
Mongdu	121	30.3
Racidong	69	17.3
Buthidong & others	210	52.4
Age (mean ± SD)	25.53 years	± 6.34
Educational status		
No education	207	51.8
Primary incomplete	107	26.8
Primary and above	86	21.6
Occupational status		
Housewife	312	78.0
Professional	88	22.0
Amount of land owned in Myanmar (mean ± SD)	5.27 acres	± 6.22
Number of children (mean ± SD)	3.98	± 2.60
Listening to radio		
Yes	233	58.2
No	167	41.8
Internet use		
Yes	103	25.8
No	297	74.2
Prime source of FP knowledge (N = 335)		
Doctor/nurse	154	38.5
NGO/health worker	181	45.3
Person(s) who make SRH-related decisions		
Wife and husband	272	68.0
Husband	128	32.0

### Different contraceptive methods heard and used by Rohingya women

Table 2 presents data on contraceptive methods that respondents had heard of and currently used. Of the Rohingya refugee women, 195 (48.7%) heard about condoms, however, only 8 (2%) of their husbands used them during the survey period. Moreover, 336 (84%) were aware of the oral contraceptive pill (OCP) and 115 (28.8%) were using it. In addition, only 42 (10.5%) heard about intra-uterine devise (IUD), 9 (2.3%) were aware of Norplant as the contraceptive, but no one had used either of the two methods. Furthermore, 356 (89%) knew about the injection Depot-Provera and 162 (40.5%) had used it during the survey.

**Table 2:** Different family planning methods cited and used by the Rohingya women

Contraceptive method	Heard about a method		Use of contraception	
	Yes [N (%)]	No [N (%)]	Yes [N (%)]	No [N (%)]
Condom	195 (48.7)	205 (51.3)	8 (2.0)	392 (98.0)
Oral contraceptive pill (OCP)	336 (84.0)	64 (16.0)	115 (28.8)	285 (71.2)
Intrauterine device (IUD)	42 (10.5)	358 (89.5)	0 (0.0)	400 (100)
Norplant	9 (2.3)	391 (97.7)	0 (0.0)	400 (100)
Injection depot-provera	356 (89.0)	44 (11.0)	162 (40.5)	238 (59.5)
Female sterilization	14 (3.5)	386 (96.5)	13 (3.3)	387 (96.7)

### Reasons for not using FP by Rohingya women

**Fig. 1:**
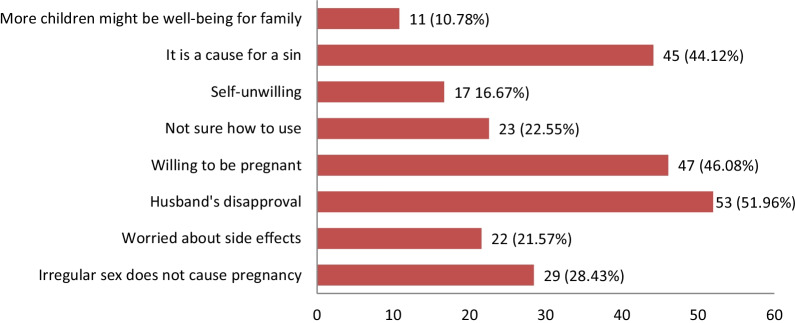
Reasons for not using contraceptive by the Rohingya women (N = 102)

Figure 1 displays the distribution of the causes for not adopting contraceptive measures among the respondents (N = 102) who were given the option. More than half of them, 53 (51.96%), acknowledged that they were not using the family planning method due to their husbands’ disapproval; 47 (46.08%) were not using it as they wanted to get pregnant; 45 (44.12%) felt that using the FP method was considered as a sin; 29 (28.43%) thought that irregular sexual intercourse was a way to avoid pregnancy; 23 (22.55%) did not know how to use a contraceptive; 22 (22.57%) were worried about probable side effects; 17 (16.67%) did not want to use any; 11 (10.78%) believed that more children might bring financial solvency to the family; and 7 (6.86%) respondents felt that contraceptive usage would reduce the pleasure of sexual intercourse.

### Rohingya women’s access to FP programs and services

**Fig. 2:**
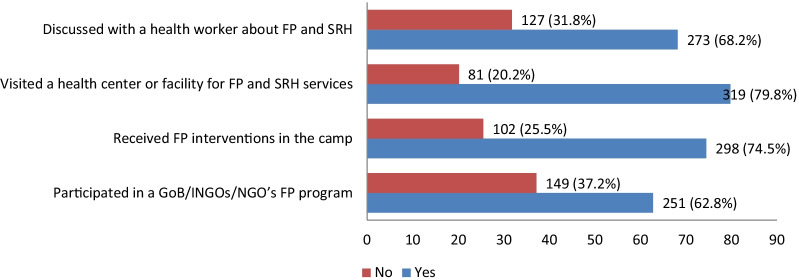
Rohingya women’s access to FP and RH services

Figure 2 illustrates the respondents’ access to health services and participation in different FP-related programs. Of the 400 Rohingya refugee women, 62.8% reported participating in a FP related meeting or event organized by GoB/INGOs/NGOs, and almost three-quarters (74.5%) received FP-related interventions of government and NGOs in the camp. Furthermore, about 80% of the study participants visited a health center or facility due to FP and 68.3% talked with a health worker about FP and SRH issues.

### Rohingya women’s FP knowledge

**Fig. 3:**
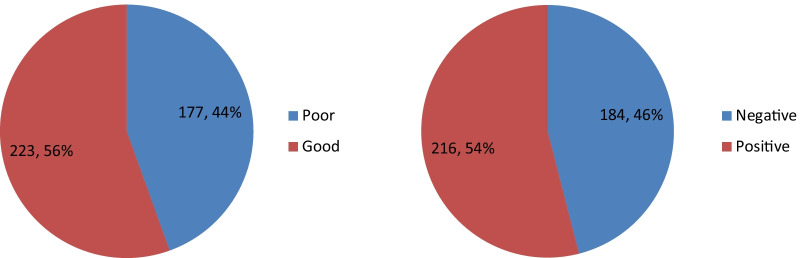
Distribution of the study participants’ level of FP knowledge (left) and FP attitude (right)

Percentages with mean scores of Rohingya women’s FP knowledge-related items are reported in Table 3. Of the 400 respondents, 180 (45%) were aware of the appropriate age of marriage for a girl. Only 162 (40.5%) respondents answered correctly whether taking a permanent contraceptive has any physical harm. Regarding whether contraceptive use had a negative effect on the husband-wife sexual relationship, 45.5% of respondents had appropriate information. Moreover, 63% responded correctly regarding the consequences of unintended or unplanned pregnancy. In addition, two-thirds of the participants (66.5%) answered correctly that there might be a risk for a woman if she has two births in a period of less than 2 years. In addition, Fig. 3 depicts that 223 (56%) respondents had good knowledge regarding FP.

**Table 3:** Descriptive analysis of FP knowledge-related items of the Rohingya women

Item	Definitely false/probably false N (%)	Don’t know N (%)	Definitely true/probably true N (%)
A girl can be married before 18 years old	212 (53.0)	8 (2.0)	180 (45.0)
A couple can limit their family by using FP	10 (2.5)	16 (4.0)	374 (93.5)
If any couple does not adopt any FP method they have a risk for unintended pregnancy	18 (4.5)	67 (16.8)	315 (78.8)
Taking an oral pill makes periods regular	10 (2.5)	76 (19.0)	314 (78.5)
There is no physical harm for those who take a permanent contraceptive	175 (43.8)	63 (15.8)	162 (40.5)
Taking contraceptives has a negative effect on the husband-wife sexual relationship	182 (45.5)	70 (17.5)	148 (37.0)
Unintended or unplanned pregnancy might lead to unsafe abortion	19 (4.8)	129 (32.3)	252 (63.0)
A woman might have a risk if there is less than 2 years between two births’	22 (5.5)	112 (28.0)	266 (66.5)
A wife is responsible for giving birth to a female child	277 (69.3)	37 (9.3)	86 (21.5)
Use of condom might protect from STDs like AIDS	6 (1.5)	104 (26.0)	290 (72.5)

### Rohingya women’s FP attitude

Table 4 shows that only 159 (39.8%) Rohingya refugee women agreed that having two children is enough for a couple. Besides, 120 (30%) thought that using FP might be regarded as a sin, and slightly less than one-quarter (23.3%) believed that discussing FP with their husband might lead to sin. In addition, 272 (68%) believed that one should not use FP if her husband objects. Of the study participants, 57% supported the idea to bear children until a male child is born and 40% of them would express more happiness if a male child is born. More than half (52%) of the respondents agreed that having more sons would ensure a more secure life for parents in elderly age. Moreover, 216 (54%) respondents had a positive attitude towards FP.

**Table 4:** Descriptive analysis of FP attitude-related items of the Rohingya women

Item	Strongly agree/agree N (%)	Neural N (%)	Strongly disagree/disagree N (%)
Having two children is enough for a couple	159 (39.8)	108 (27.0)	133 (33.3)
Using FP might be considered a sin	120 (30.0)	40 (10.0)	240 (60.0)
A couple should discuss and plan the timing of having a baby	302 (75.5)	36 (9.0)	62 (15.5)
Discussion of FP with husband might be a considered a sin	93 (23.3)	23 (5.8)	284 (71.0)
One should not adopt FP if her husband has an objection to it	272 (68.0)	35 (8.8)	93 (23.3)
One should keep having children until a male child is born	228 (57.0)	21 (5.3)	151 (37.8)
I might get more food cards if I have more children	29 (7.3)	103 (25.8)	268 (67.0)
I am happy if the newborn is a son	153 (38.3)	12 (3.0)	235 (58.8)
Having more sons will bring more security to parents in their elderly age	196 (49.0)	12 (3.0)	192 (48.0)
Having more daughters might be a burden	52 (13.0)	32 (8.0)	316 (79.0)

### Rohingya women’s FP practice

Table 5 showed that 43% of the respondents reported that they always felt ashamed to discuss FP and 45% were usually afraid of FP discussions with their husbands. In addition, about one-quarter felt shy while discussing FP with relatives and neighbors. About three-quarters of Rohingya refugee women regularly used contraceptives during the survey period. Furthermore, 60% of the respondents regularly obtained new contraceptives after running out of them and 62 percent continued FP use despite experiencing side effects.

**Table 5:** Descriptive analysis of FP practice-related items of the Rohingya women

Item	Never/Rarely N (%)	Sometimes N (%)	Often/Always N (%)
I feel ashamed to discuss FP with my husband	227 (56.8)	49 (12.3)	124 (31.0)
I am afraid of discussing FP with husband	222 (55.5)	62 (15.5)	116 (29.0)
I discuss FP with relatives and neighbors	111 (27.8)	201 (50.3)	88 (22.0)
I feel ashamed to discuss FP with relatives and neighbors	249 (62.3)	50 (12.5)	101 (25.3)
I use FP method(s)	105 (26.3)	25 (6.3)	270 (67.5)
I collect FP materials after finishing	161 (40.3)	68 (17.0)	171 (42.8)
I am satisfied using FP	111 (27.8)	43 (10.8)	246 (61.5)
I can accept FP side effects	147 (36.8)	127 (31.8)	126 (31.5)
I discuss FP-related issues with my husband	111 (27.8)	68 (17.0)	221 (55.3)
I inform the health worker if I feel any complexities related to FP	113 (28.3)	43 (10.8)	244 (61.0)

### Influencing factors associated with FP-related KAP

Table 6 demonstrates that Racidong in Myanmar as the region of residence (β = 0.09, t = 2.84, p = 0.005), having a profession (β = 0.10, t = 2.73, p = 0.007), having less children (β = − 0.28, t = − 7.28, p < 0.001), having a physician/nurse as the source of FP knowledge (β = 0.21, t = 6.45, p < 0.001), having GoB/INGOs/NGOs’ FP interventions at the camp (β = 0.15, t = 3.62, p < 0.001), visiting a clinic/health facility (β = 0.22, t = 4.96, p < 0.001), and talking with any health care provider (β = 0.24, t = 5.54, p < 0.001) were significantly associated with Rohingya women’s better knowledge on FP and accounted for 66% of the variation in this regard.

**Table 6:** Linear regression analysis of factors associated with FP knowledge, attitude and practice of the Rohingya women

Variable	Knowledge on FP			Attitude towards FP			Practice of FP		
	β	t	p	β	t	p	β	t	p
Region of residence in Myanmar^a^	0.09	2.84	0.005	0.00	0.12	0.902	0.07	2.57	0.010
Educational status^b^	0.02	0.67	0.507	0.02	0.37	0.713	− 0.08	− 2.71	0.007
Occupational status^c^	0.10	2.73	0.007	− 0.02	− 0.38	0.703	0.07	2.27	0.024
Age^d^	0.03	0.84	0.399	− 0.06	− 1.33	0.183	0.02	0.58	0.565
Amount of land owned in Myanmar^d^	0.04	1.33	0.185	0.11	3.02	0.003	0.03	1.23	0.220
Number of children^d^	− 0.28	− 7.28	< 0.001	− 0.17	− 3.83	< 0.001	− 0.15	− 4.54	< 0.001
Persons(s) who make SRH decision^e^	0.06	1.83	0.068	0.06	1.49	0.138	0.04	1.44	0.150
Prime source of FP knowledge^f^	0.21	6.45	< 0.001	0.18	4.88	< 0.001	0.13	4.66	< 0.001
Having had FP interventions in the camp^g^	0.15	3.62	< 0.001	0.25	5.45	< 0.001	0.10	2.81	0.005
Having participated in FP program^g^	0.05	1.39	0.164	0.12	2.82	0.005	0.07	2.33	0.020
Having visited clinic/health facility^g^	0.22	4.96	< 0.001	0.19	3.78	< 0.001	0.46	12.31	< 0.001
Having talked with health care provider about FP/RH^g^	0.24	5.54	< 0.001	0.16	3.19	0.002	0.24	6.32	< 0.001
	R^2^ = 0.66	F = 64.84	< 0.001	R^2^ = 0.56	F = 59.56	< 0.001	R^2^ = 0.74	F = 170.36	< 0.001

^a^1 = Mongdu/Racidong, 2 = Buthidong

^b^1 = No education, 2 = Have education

^c^1 = Housewife, 2 = Professional

^d^Continuous variable

^e^1 = Husband & wife, 2 = Husband

^f^1 = Others, 2 = Physician/nurse

^g^1 = No, 1 = Yes

Furthermore, the amount of land owned in Myanmar (β = 0.11, t = 3.02, p = 0.003), having less children (β = − 0.17, t = − 3.83, p < 0.001), having a physician/nurse as the source of FP knowledge (β = 0.18, t = 4.88, p < 0.001), having GoB/INGOs/NGOs’ FP interventions in the camp (β = 0.25, t = 5.45, p < 0.001), participating in a FP awareness program (β = 0.12, t = 2.82, p = 0.005), visiting a clinic/health facility (β = 0.19, t = 3.78, p < 0.001), and talking with a health care provider (β = 0.16, t = 3.19, p = 0.002) contributed significantly to the regression model (F = 59.56, df = 4/387, p < 0.001) and appeared as predictors of Rohingya women’s more positive attitude towards FP and accounted for 56% of the variation of the outcome variable.

It was also found that, having resided in Racidong in Myanmar before coming Bangladesh (β = 0.07, t = 2.57, p = 0.010), having a profession (β = 0.07, t = 2.27, p = 0.024), having less children (β = − 0.15, t = − 4.54, p < 0.001), having a physician/nurse as the source of FP knowledge (β = 0.13, t = 4.66, p < 0.001), having GoB/INGOs/NGOs’ FP interventions in the camp (β = 0.10, t = 2.81, p = 0.005), participating in a FP awareness program (β = 0.07, t = 2.33, p = 0.020), visiting a clinic/health facility (β = 0.46, t = 12.31, p < 0.001), and talking with a health care provider (β = 0.24, t = 6.32, p < 0.001) were the most important factors influencing a more regular, healthy practice of FP and accounted for 74% variations of good FP practice.

## Discussion

This study assessed Rohingya refugee women’s knowledge, attitude, and practice towards FP along with the overall status of FP in the camps. Our study found that despite their familiarity with the traditional contraceptives like injections, oral pills, and condoms, most of the respondents are not familiar with the modern contraceptive methods such as IUDs and Norplant. According to local media reports, Rohingya refugee women would take oral pills given by health stations in the camps and would throw them away upon returning home. Later, when they were given the 3-month injection method they accepted it. Although contraceptive methods have been introduced among the Rohingyas who took shelter in Bangladesh since the 1990s, recently FP programs increased after a massive influx of Rohingyas into the country. Indeed, NGOs do not disseminate the information of different modern birth control methods among the Rohingya women as the women are reluctant to use them [3, 26, 33].

Consistent with other studies [34, 35], our findings also demonstrate that there is a dearth of accurate and sufficient knowledge of FP among the Rohingya women living in the refugee camp of Cox’s Bazar even though they have some ideas about FP and using contraceptives. Even for women who report to be willing to pursue FP approaches, discontinuation of use may be motivated by a general feeling of uncertainty and fear, particularly about health-related side effects [4]. Away from the positive knowledge gained through education, this group is influenced by traditional religious practices [33].

Half of the respondents lack proper knowledge of whether a girl is eligible for marriage before the age of 18. Among the Rohingyas, girls are likely to get married at an early age. A previous study [4] noted a clear preference for girls but not boys for child marriage. There are some reasons behind early marriage of girls in Rohingya society [1, 4]. Firstly, this tendency is more prevalent in families with more daughters because parents feel that more than one daughter still living with the parents is a burden, and older parents want all their daughters to get married while they are still alive. Secondly, members of the community also say different types of harsh words and pass nasty comments if more than one young girl lives with them in the household, so, the parents want to marry their daughters off as early as possible. Thirdly, as is prescribed by their faith, girls are deemed suitable for marriage until they hit puberty. Parents believe that keeping young girls unmarried at home for a long time is a sin. Fourthly, the financial insolvency of Rohingya people leads them to send their daughters to the in-laws’ house so that they do not have to bear their living costs for too long a period. Ainul et al. [4] identified some important shifts in the trends and behaviors of marriage among Rohingya refugee after displacement. Unlike Myanmar, the camps in Cox’s bazar have no age limit for marriage, consequently, Rohingya girls and boys tie the knot as early as the age of 14/15 years.

The present study found that Rohingya women have also shown interest in having more children. Our finding is supported by a previous study [16]. Lagging in their education, they still see childbirth as an achievement [36]. Half of the respondents think having more children will give them more protection and support in their old age. They believe that children are a God-given blessing and they will receive more rewards or benefits if they have more children. Getting food cards is also a factor in the camps since it is allocated to every child [37]. By showing that card, parents get various benefits, including food, medicine, and clothes. They know that they will get more food cards or help if they have more children [38]. Many of the children's food items they get with food cards are sold outside the camp for money. During the study it was also found that at the Teknaf bus station food items provided by the UN were being sold openly among the host community and tourists. Therefore, a cohort of the Rohingya families does not use contraceptives, although they are urged by the government to practice this FP method. Another reason the Rohingya population has more children may be explained by their thinking of the Myanmar government’s oppression to eradicate them ethnically [39, 40]. Having more children can also be an attempt to sustain their existence as a nation. This assumption is also supported by media reports [40].

Our study findings also showed that more than two-thirds of the Rohingya women thought family planning methods should not be used without their husband’s approval. In Rohingya society, patriarchy prevails and women mostly obey their husbands as they regard it as a sin to do anything without their husband’s permission. Therefore, the use of CPR is low due to the husbands’ reluctance for their wives to use contraceptives [41]. In addition, according to our findings, 58% of respondents said that they should continue childbearing until the birth of a son. Besides, 40% of the respondents said that having a son is a matter of pride, whereas one-fifth attributed having daughters as a burden. Parents also have a similar feeling as arranging the marriage of a daughter costs a lot, and daughters would not be responsible for taking care of their parents in the future. On the contrary, a male child is highly desired by the Rohingya couples, as the think that boys can earn money and will be responsible for taking care of their parents later in life.

Our data also found that more than 40% were ashamed of and afraid of discussing FP with their husbands, considering it a sin. In Rohingya society, FP or birth control are perceived as a high-level taboo. Rohingya women are typically conservative due to their religious and social values. There is no positive viewpoint regarding FP or birth control in Rohingya society, and religiously it is considered an immoral behavior [29]. Those who have not used FP yet and are still reluctant to use it might be regarded as being extremely against FP. This type of people is called the hard-core resister group by Rogers [42]. A typical couple in this category would be very religious and the husband an older religious leader. A strategic communication program would need to be implemented in order to make them more open to FP methods. If nothing is done to deal with the KAP of this radical group, then it is likely that they will contribute to significant population growth in the refugee camps.

Comparing Rohingya women from the surveyed areas, the knowledge and behavior of the women from Rachidong area are better than those of the women from Maungdaw and Buthidong area as the transportation system in Rachidong is better and Rachidong people have more opportunities of commuting to the city for study and work.

According to the results, Rohingya women involved in various professions had a better KAP of FP. They usually work with various NGOs serving as the teacher for providing education and psychosocial support, community mobilizers for nutritional activities, cleaners, or day laborers. NGOs offer different training and awareness sessions for them, so their attitudes and behaviors towards FP are more positive. They are also interested in learning new things and have a better chance to communicate with the Bangladeshi staff more closely.

Women with fewer children were found to have better FP-KAP in our study. This cohort is more conscious and progressive than others as they engage and remain focused actively in various awareness programs. Consequently, they become the primary and early receivers of FP services. Family members, particularly husbands and mothers-in-law, play a key role in making decisions about a married girl’s childbearing and contraceptive options in Rohingya society [4]. Nevertheless, Rohingya women who can make their own decisions about their health have better FP-KAP. Generally, these women are more aware and self-reliant. They also have a better attitude and perspective since their husbands and families allow them to express their views independently.

We observed that the Rohingya women who received a consultation from doctors and nurses had better FP-KAP. In this case, the women’s interest in FP plays a significant role in listening carefully to the information provided by health care providers and applying it in real life. Health care providers have been able to talk to them, change their attitudes and make them regard FP in a more positive way. According to the Department of Family Planning, besides raising awareness of birth control attitudes among the Rohingya men and women, doctors and nurses working in clinics and health facilities also provide various suggestions and medicines for pregnancy, maternity, child health, and general health services. Such efforts are more significant than those of NGO health workers. Many Rohingya couples now do not want to have 10–12 children; instead, they want to limit the number of children to 4–5 [36]. Most of these programs and services have created a positive outlook on FP that makes women and girls more aware and engaged on the topic than before [33].

The Rohingya women who had visited a clinic and talked to a doctor were more likely to have better FP-KAP. Doctors and nurses play a supporting role in understanding FP. Visiting a clinic, they can observe the posters and communication materials and can be informed about different aspects of FP and maternal health issues.

The study has some limitations. Firstly, the data from the participants may have been influenced by social desirability, which could affect the validity of the outcome. Secondly, this analysis could provide a more precise understanding and a more in-depth insight if qualitative data were collected. Thirdly, the data was collected from only one camp due to inadequate research funds.

## Conclusion

The study showed that the comprehensive FP-KAP capability of Rohingya refugee women was low. Contraceptives among the Rohingyas were unpopular, mainly due to a lack of general education and awareness of family planning. In addition, family planning initiatives among Rohingya refugees were limited by various traditional cultural and religious beliefs. Participation in the FP program, visiting a health facility, and talking with a health care provider were reported as the most significant predictors for a better FP-KAP. Therefore, designing appropriate campaigns and developing effective communication materials is important to improve this vulnerable community’s maternal health status. Accordingly, politicians, program managers, and implementers should educate and equip Rohingya women on essential FP, SRH, and maternal health-related topics through a sustainable and continuous training program. Moreover, the program should involve religious leaders in planning and implementations phases, and provide them with appropriate training so that they can play a supportive role as community leaders.

## Data Availability

All of the primary data has been included in the results. Additional materials with details may be obtained from the corresponding author if required.

## Abbreviations

ANOVA: Analysis of variance
FP: Family planning
GoB: Government of Bangladesh
ICDDR,B: International Centre for Diarrhoeal Disease Research, Bangladesh
INGOs: International non-governmental organizations
ISCG: Inter Sector Coordination Group
IUD: Intrauterine device
KAP: Knowledge, attitude and practice
NGOs: Non-governmental organizations
OCP: Oral contraceptive pill
SD: Standard deviation
SPSS: Statistical Package for Social Sciences
SRH: Sexual and Reproductive Health
UNICEF: United Nations Children’s Fund
UNHCR: United Nations High Commissioner for Refugees
UNFPA: United Nations Population Fund
WHO: World Health Organization
